# ‘This town’s a different town today’: Policing and regulating the night-time economy

**DOI:** 10.1177/1748895809343409

**Published:** 2010-03

**Authors:** Phil Hadfield, Stuart Lister, Peter Traynor

**Affiliations:** University of Leeds, UK

**Keywords:** night-time economy, police, alcohol, licensing, anti-social behaviour

## Abstract

This article considers recent policing and regulatory responses to the night-time economy in England and Wales. Drawing upon the findings of a broader two-year qualitative investigation of local and national developments in alcohol policy, it identifies a dramatic acceleration of statutory activity, with 12 new or revised powers, and several more in prospect, introduced by the Labour Government within its first decade in office. Interview data and documentary sources are used to explore the degree to which the introduction of such powers, often accompanied by forceful rhetoric and high profile police action, has translated into a sustained expansion of control. Many of the new powers are spatially directed, as well as being focused upon the actions of distinct individuals or businesses, yet the willingness and capacity to apply powers to offending individuals in comparison to businesses is often variable and asymmetrical. The practice of negotiating order in the night-time economy is riddled with tensions and ambiguities that reflect the ad hoc nature and rapid escalation of the regulatory architecture. Night-time urban security governance is understood as the outcome of subtle organizational and interpersonal power-plays. Social orders, normative schemas and apportionments of blame thus arise as a byproduct of patterned (structural) relations.

## Introduction: Urban spaces and the night-time economy

The current debate on anti-social behaviour and violence in our urban environments is but the latest in a long tradition of public disquiet over the urban condition. Over the last decade, the night-time economy (NTE) and its regulation, management and policing has emerged as a key focus for urban public policy, reflecting the changing character of urban sites, particularly the rise of a new alcohol-fuelled, consumption-driven, night-time high street (Hadfield, 2006; Hough and Hunter, 2008). The transformation of many urban centres has been remarkable, with the commercial and civic remnants of past economic eras having morphed into ‘themed’ pubs and ‘designer’ bars and clubs, thus mirroring the ubiquitous chain stores, designer boutiques and franchised coffee shops of daylight consumption. These licensed premises are often clustered into easily identifiable zones, notable for their youth-orientation and focus upon alcohol consumption as key social activity, economic driver, and cultural motif.

As a result of these environmental characteristics, nightlife zones are contested spaces. Social disorders, such as public drunkenness—observable as stable patterns of socially proscribed behaviour amongst the street population—elicit very different perceptions and emotions among different groups of people (Innes, 2004; Sampson, 2009). Whether observed through direct experience, or vicariously through local gossip and media filtration, these dramas of intoxication signal fun and adventure for some, and danger, stress and anxiety for others (Thomas and Bromley, 2000). Given the perennial attractions of being ‘where the action is’ (Goffman, 1967), Britain’s major nightlife areas routinely entice up to 100,000 revellers, a transient population whose footfall places severe stresses on extant modes of governance within these sites of consumption.

In response to the pressures associated with the NTE, novel attempts to develop and sustain ‘partnership’ working are emerging from an increasingly variegated mix of agencies including police, local authorities, health trusts, the licensed trade, security companies, residents groups, and charitable/voluntary agencies. Since November 2005, these activities have been orchestrated partly in accordance with the four statutory objectives of the Licensing Act 2003: the prevention of crime and disorder, the promotion of public safety, the prevention of public nuisance and the protection of children from harm. The importance of this legislation cannot be underestimated as it has inserted the control of alcohol into the heart of urban governance (see, for example, Department for Communities and Local Government, 2009: 20).

The pairing of situational crime prevention technologies with various forms of spatially-targeted powers that threaten restrictions on the presence, behaviour, and movement of individuals and groups, is now ubiquitous in British towns and cities, as in other cultural contexts and jurisdictions (Hadfield, 2009a). This intense activity has been informed by the realization that private security governance, as performed by pubs and clubs, is necessary, but insufficient to tackle the problems of (dis)order generated within these social milieux (Hadfield, 2008). Attempts to exert control over night-time spaces often find themselves out-of-step with the culture of excess prominent in many peoples’ drinking practices (Martinic and Measham, 2008; Measham and Brain, 2005; Room, 2007). They may also face resistance from those businesses who supply, and in many cases help generate, such demands. As such, the normality of anti-social behaviour within the NTE raises dilemmas of governance in terms of the limits of regulation and control (Garland, 1997).

This article explores contemporary state responses to the NTE as a regulatory dilemma. It begins by tracing the recent history of police campaigns and new legislation which involve major, prima facie, intensifications of state control. Various civil/criminal and public/private mechanisms for regulating public and quasi-public space are then described and analysed on the basis of the findings of a recent qualitative research project on local and national alcohol policy.

## Controlling the night: A decade of grip tightening

Toward the end of the 1990s, pronounced rises in recorded violence and criminal damage were correlated with temporal and spatial patterns of economic development within the NTE (Hobbs et al., 2003; Home Office, 2001; Warburton and Shepherd, 2004). In combination with major reforms to the licensing laws, introduced through the implementation of the 2003 Act, a highly politicized context emerged in which public, media and professional concerns regarding alcohol-related crime and disorder (and the health implications of national drinking patterns) conspired to create a crisis of legitimacy for the British state. As a consequence, numerous concessions in the form of new powers and funding for police and local authorities were made to counterbalance or reconfigure what had been a thoroughly neo-liberal policy agenda in which the alcohol industry appeared to have ‘captured’ the means of their own regulation (Hadfield, 2006; Room, 2004). Subsequently, a series of Home Office funded enforcement campaigns were orchestrated between 2004 and 2007 under the auspices of the Alcohol Misuse Enforcement Campaign (AMEC), the Tackling Underage Sales of Alcohol Campaign (TUSAC) and the Responsible Alcohol Sales Campaign (RASC) to tackle alcohol-related disorder. These police-led campaigns were augmented by a significant ‘ramping up’ of formal police powers and (administrative) regulatory options, which provided the authorities with more diverse methods and sanctions aimed at regulating the (mis)conduct of individuals but also the ‘risky’ operational practices of businesses profiting from the sale of alcohol (both at on and off-licensed premises), for example, the selling of alcohol to underage drinkers or those already drunk. Hence the regulatory drive was aimed at those on both the demand-side and the supply-side of the market in alcohol sales, with the latter being held culpable for the often intoxicated and sometimes disorderly behaviour of the former.

Table 1 variously categorizes the main interventions into one of three forms: those focusing on specific persons, specific places (the subject of which may be individuals or groups occupying particular locations, or businesses operating within such locations), or individual licensed venues. Many of the powers are notable for their risk-based preventative logic that functions through processes of surveillance, identification, categorization, and exclusion. For example, the (licensing) powers to close premises temporarily in order to tackle disorder can be both reactive and anticipatory. Further civil injunctions are, of course, available to local authorities in relation to noise nuisance. Whilst these powers overlap, to some degree, with those intended to address alcohol-related disorder, they remain beyond the scope of this article.

**Table 1.** Recent legislative powers and sanctions relevant to tackling crime and disorder in the night-time economy in England and Wales

*Nature*	*Type*	*Enabling legislation*	*Power / Sanction*
Person-based	Anti-Social Behaviour Orders	Crime and Disorder Act 1998, s.1	Civil orders widely used to exclude persons from public space, including night-time drinking areas
	Penalty Notices for Disorder	Criminal Justice and Police Act 2001 s.1	Summary fines which police and accredited persons can issue for a range of low-level disorder offences, often associated with alcohol consumption
	Drinking Banning Orders	Violent Crime Reduction Act 2006 s.1-14	A civil order excluding ‘risky’ individuals from licensed premises within a defined geographical area
Place-based	Directions to Leave a Locality	Violent Crime Reduction Act 2006 s.27	Police can require persons to leave a specified locality if that person is judged likely to contribute to alcohol-related crime and disorder
	Dispersal Orders	Anti-Social Behaviour Act 2003 s.30-36	Police can exclude groups of two or more persons from a designated area, where their behaviour or presence is likely to be perceived by others as anti-social
	Designated Public Places Orders	Criminal Justice and Police Act 2001 s.13	Allows councils to identify public places in which the consumption of alcohol is prohibited and can be confiscated by the police
	Cumulative Impact Policies	Guidance accompanying the Licensing Act 2003	Allows for a refutable presumption against the granting of new Premises Licences, or variations to licences so as to extend opening hours, within a given area
	Alcohol- Disorder Zones	Violent Crime Reduction Act 2006 s.15-20	Allows licensing authorities to design an action plan to remedy alcohol-related problems within a specified area
Venue-based	Licensing Conditions	Licensing Act 2003	Allows licensing authorities to specify how premises will be run, including the required introduction of various crime prevention measures
	Licence Review	Licensing Act 2003 s.51	Allows a responsible authority or an ‘interested party’ to request a review of the licence conditions
	Licensing Enforcement Powers	Licensing Act 2003 Pt. 7	Restates the offences of supplying alcohol to a) under-age drinkers and b) those deemed to be drunk, and allows authorities to check compliance by test-purchases
	Closure Powers	Licensing Act 2003 Pt. 8	Allows police to close temporarily certain premises, or all premises in a specific area, where there is actual or anticipated disorder, or to abate noise-related nuisance

## Research design

In exploring the implications of the powers within this list, this article draws on the findings of a two-year research project which combined an intensive twin-site case study with broader explorations of the national dimensions of alcohol policy formulation and delivery. Local contexts and actions were explored in a 715,000 population city in the North East of England and a geographically dispersed, 335,000 population, metropolitan district comprising several small to medium sized towns, in the North West of England. Fifty in-depth interviews were conducted in these sites (25 in each) between June 2007 and December 2008, with a further seven local residents participating in a focus group at the latter location. The locations were selected in order to compare different local contexts and to explore elements of their specificities or convergences of approach in response to central government dicta. Interviewees were selected purposively to encompass representatives of all the major and more minor regulatory players: Crime and Disorder Reduction Partnerships (CDRPs), police, council licensing departments, social services, health and treatment services (statutory and voluntary), education and young peoples’ services, government regional offices, Chambers of Commerce, drinks retailers, residents’ groups and voluntary sector associations.

To encompass the national policy arena and its interplays with the local, a further 20 interviews were conducted with prominent figures in central and local government, the police, health care, the legal system, the drinks and leisure industries, and a range of professional and non-government organizations, including charities and pressure groups. Interviews were semi-structured around established and emergent themes and sought to explore the views of those who shape various facets of national alcohol policy and its interconnections with local policy development and delivery. A range of stakeholders in the research sites and nationally were asked to reflect upon the rationale and implementation of the various powers. Some of the views expressed related to powers already implemented, while other, yet-to-be introduced measures, were judged in prospect.

## A new era of nightlife governance

The various new or enhanced powers shown in Table 1—all introduced since 1998—have intensified but also re-orientated the nature of state control within the NTE, drawing together webs of actors as regulator and regulated deploying different levers of control and compliance. Until New Labour’s first term of office in 1997, long-established governmental structures and responses prevailed in which police, licensing magistrates, local authorities, and leisure businesses played distinct and largely non-integrated roles. Public policing methods were predominately characterized by the ‘fire brigade’ model of reactive vehicle-based response and the ‘swarming’ of crime scenes (Hobbs et al., 2003), while licensing operated as a largely distinct administrative technology for regulating alcohol sales (through the magistracy) and public entertainment in the form of ‘music and dancing’ (through local authorities). The Licensing Act 2003 served to intertwine these governmental fields, bringing both activities into the remit of a single licensing instrument (the Premises Licence) and single jurisdiction (the local authority licensing committee). Urban security governance thus became explicitly linked to control over the availability of alcohol and its conditions of sale, and to a tacit censorial role in the regulation of associated cultural activities (Hadfield and Measham, 2009).

As local authorities also hold jurisdiction over land use planning for leisure venues they are, in principle, able to control the NTE in both form and content (trading hours, physical design and capacity, managerial methods, licensable activities, spatial distribution). Yet the various mechanisms of procedural justice, de-regulatory checks and balances, appeal and precedent built into the planning and licensing systems significantly restrain local government action, establishing more equitable power relations between regulator and regulated (Hobbs et al., 2003; Roberts, 2009) Subsequently, the police, rather than local authorities, have arguably gained the greater leverage over the governance of the night in recent years. The Licensing Act 2003 allocated police a key role as ‘responsible authorities’ within the licensing process, with the power to make ‘representations’ to the licensing committee regarding licence applications, to instigate reviews, and propose mandatory conditions. It is an oft-overlooked fact that in the absence of representations from responsible authorities or interested parties licensing committees are not at liberty to deny new Premise Licence or licence variation applications. Thus Cumulative Impact Policies, for example, become operable only upon receipt of such representations, with responsible authorities (primarily police) also prominent in providing much of the evidential bases (local crime and public nuisance data) for their initial development.

### Controlling space/controlling persons

The recent growth of highly discretionary police powers was widely welcomed by practitioners concerned with securing order and safety in the NTE. While these powers were seen to provide the police with greater options and flexibility when asserting authority among night-time denizens, their use appears to be inconsistent owing to concerns over implementation difficulties, but also the extent to which they address in any meaningful way the nature of the problematic behaviour. A case in point were Penalty Notices for Disorder (PNDs), which can be issued for a variety of offences that may arise in nightlife areas, from being disorderly whilst drunk in a highway, other public place, or licensed premises, to buying or attempting to buy alcohol for consumption by a person under 18 in licensed premises, or urinating in a public place. Although known as ‘on-the-spot-fines’ and designed *inter alia* to reduce police bureaucracy by enabling the police to issue a swift, summary punishment (see Young, 2008), they were regarded by police at one of our sites as strictly a case disposal option for the custody suite following arrest, and often only after the ‘offender’ had spent a night in the cells to sober up. This approach was adopted in light of official guidance issued to the police that suggested initial aspirations for rapid, street-based disposal may be unworkable as ‘a penalty notice will not be appropriate where the suspect is unable to understand what is being offered to them, for example … where the suspect is drunk or under the influence of drugs’ (Home Office, 2005: 19, para 7.3). Hence, although section 5 Public Order and ‘drunk and disorderly’ offences account for 60 per cent of all PNDs (Ministry of Justice, 2008), their use in the NTE is at least partially constrained by other concurrent demands on the custody suite as well as street policing, more broadly.

Spatially-focused, person-directed approaches raised similar concerns, specifically surrounding the intoxication and hence mental incapacity/vulnerability of the target groups. For example, what might happen to a drunken person if they were ordered by the police to leave an area and possibly their friends? Likewise, as Crawford and Lister (2007) discuss in relation to dispersal orders, there were also concerns that place-based powers may merely displace disorder, particularly owing to the recidivist nature of many ‘problem drinkers’:

you can confiscate alcohol from people all day long, but if you don’t address the issues around that, then you’re just gonna be doing it every day … unless you actually try and look at ways of preventing them re-offending, you’re not gonna solve your problem. (Police Sergeant)

Concerns over the failure of such interventions to address the underlying causes of alcohol-related crime and disorder were aggravated by a lack of data sharing associated with discretionary and summary disposals, which prevented offenders’ details from being matched against those held by partner agencies. This constraint was regarded as unhelpful in preventing the identification of patterns of offending, the engagement of alcohol arrest referral schemes and other support and treatment services.

Interestingly and perhaps—as will become apparent later—significantly, Drinking Banning Orders (DBOs) were more positively received. Our interviewees regarded them as an important aid in supporting the civil powers of exclusion invested in licensees who are more directly and proximately responsible for governance within the publicly accessible, but privately owned spaces of pubs, bars and nightclubs. Partnership mechanisms such as the local trade-led ‘Pubwatch’ forums—in which licensees and police share information and cooperate in crime prevention initiatives—were instrumental in this respect. Acting as ‘knowledge brokers’ (Ericson and Haggerty, 1997), police circulated photographs of known offenders to all Pubwatch members which were then recorded in albums and displayed on montages behind the bar. Registered under the Data Protection Act, Pubwatch members were also able to generate their own intelligence for distribution among their network and with the police. In one area, the local Pubwatch had authorized the police to issue a banning order which excluded alcohol-related offenders from *all* their members’ licensed premises. Although secondary to formal penalties, the imposition of this extra-legal sanction was reported to have had a strong deterrent effect in that the resultant deprivations spoke directly to the focal concerns of offenders, thwarting their ability to inhabit those social milieux from which the seductions of violence first emanated (Winlow and Hall, 2006):

They’re [the banning orders] effective … because people are starting to say, not, ‘how much am I gonna be fined, how long am I going to prison?’ It’s ‘am I going to be banned from the pub?’ And there’s more of an issue over not being able to get the drink down your local than there is about the other consequences … It has a big social impact on those individuals that it affects. (Police Sergeant)

### Controlling space/steering commerce

While the effectiveness of banning orders and the Pubwatch schemes that support them relies upon voluntary co-operation and co-dependency with the licensed trade, premises-directed powers represent clear attempts by the state to ‘govern at a distance’ by ‘responsibilizing’ licence holders for the control of crime and disorder in, and increasingly around (e.g. any pavement, beer garden and smoking area) their venues (see Department for Culture Media and Sport, 2007: para. 1.26). Police and licensing authorities expect that licencees and their staff should be held responsible not only for the ‘responsible’ sale of alcohol, but also for complying with many other statutory requirements and regulations (such as specific sets of licensing conditions) relating to the running of their premises, which may cover a myriad of issues, including substantial emphases on ‘security’ and ‘good neighbourliness’. Much of the police and trading standards enforcement activity that has taken place in recent years has sought to underline such expectations, wherein complying with imposed licensing conditions is seen as a means of minimizing the risks of disorder. The levers of compliance here are largely instrumental. Sanctions for non-compliance threaten the commercial viability of licensed premises, whether in the form of fines, or the financial costs borne from being required by licensing authorities to introduce (costly) crime prevention technologies, changes to profitable and established operating practices, or ultimately to close down (temporarily or otherwise) a venue.

Enforcement activity in respect of breaches of statutory requirements and licensing conditions is a matter for council officers, both from licensing and environmental departments. These officers are in regular contact with each other and with police and sometimes make ‘multi-agency’ inspection visits to premises in order, as one council licensing officer put it, ‘to advise and inform licence holders what their responsibilities are’. In both areas, data within and between agencies was collated, risks evaluated and premises categorized in the form of a ‘problem premises register’. Premises raising concerns across a spectrum of community safety issues, from high levels of drunkenness and violence, to noise escape, glass injuries, and customers slipping over on wet floors which had not been properly cleaned, were classified as ‘poorly operated’. In accordance with the principles of effective enforcement described in the Hampton Report (2005), once graded into categories of risk, those premises identified as ‘underperforming’ (i.e. as presenting a high risk of hosting crime and disorder) were then selected for further proactive surveillance, inspection and enforcement operations.

Our findings suggest that regulators are more inclined to gently steer businesses towards compliance with licensing conditions than threaten them directly, or proceed with coercive action. There was much evidence of restraint and even reticence in applying the range of powers and sanctions at their disposal. For example, the number of PNDs issued for selling alcohol to a drunken person increased between 2006 and 2007 from 47 to only 81 (Ministry of Justice, 2008), despite the Home Office placing this offence to the fore within its Responsible Alcohol Sales Campaign of 2007 (Slade, 2007). Tellingly, within one of our sites, Pubwatch members were, in many instances, warned of the timing of such blanket enforcement operations as a ‘reward’ for their cooperation in other matters. This conciliatory position was explained in terms of the fear of driving a wedge between interlocutors who had little choice but to work together, often on long-term bases. Thus, the real utility of premises-directed powers was to provide regulators with a position of strength from which to enter a process of dialogue with licensees and thereby negotiate graduated outcomes in the shadow of the law (see Hawkins, 1984; Manning, 1987). Such negotiations might typically surround the drafting of non-contractual ‘memoranda of agreement’ between the police and licensees concerning a suite of preventative security measures to be adopted. Review proceedings were regarded as a breakdown in the usual functioning of these informal, compliance-based mechanisms, occurring only where licensed operators were judged recalcitrant, recidivist (incapable of resolving serious and recurrent problems), or more rarely, openly defiant of police authority. As one police licensing officer explained, the introduction of a sliding scale of regulation was regarded as having permitted the authorities greater leverage over the actions of operators:

The Review process is really really good. Now before I used to have licensed premises taking the mick. The only thing I could do if they didn’t respond to partnership activity was go down the revocation route, which was like the nuclear option. And of course, they would fight [it] because this was their business. Well under the Review process, we can call them in, have their licence looked at, and potentially just add conditions rather than completely close them down … It’s a far more measured approach.

The previous licensing system, under the Licensing Act 1964, therefore, is presented as one in which licensing justices were understandably reluctant to countenance the imposition of a loss of livelihood upon licensees, save in the gravest of circumstances. This lack of flexibility had the effect of restricting enforcement options, allowing low-level miscreants to operate with relative impunity, as recalled by the same licensing officer: ‘I remember the frustration of it. All we ever really used to do was put an entry in the licensing register … nothing ever seemed to change’. The *threat* of Review now acts as a key determinate in achieving compliance:

Obviously they’re scared to death, the licensed trade doesn’t want to go to Review and neither do we, *this is the thing, neither do we*. But we will if we have to … And every now and again, there are serious incidents that we have to just go straight for the stick. But we won’t do so out of choice. (Police Inspector)

It is now possible, many felt, to improve operating standards in ‘problem’ premises without undertaking the detailed case work involved in Review and the protracted legal and quasi-legal wrangling elicited by any subsequent engagement with the appeal process. Chinks in the armour of enforcement continued to exist and it was typically through the trade’s engagement of specialist licensing lawyers that these might be exposed. The simple action of requesting an appeal against the licensing authority’s decision often frustrated police intentions, leading as it typically did to significant periods of delay (up to two years) awaiting a hearing date in the Magistrates’ Court. During such time, the appellants remained free to trade ‘as usual’ without having to comply with the licensing authority’s judgments.

The Guidance to the 2003 Act makes it clear that, ‘Licensing authorities should only impose conditions which are necessary and proportionate for the promotion for the licensing objectives’ (Department of Culture Media and Sport, 2007: 10.15). The question of weighing proportionality, of course, remains a grey area, but one which can raise the stakes, making appeals more likely. For example, in granting a licence variation to permit trading after midnight, a condition might be imposed that the premises employ at least three door supervisors during this time period. This additional cost might outweigh the commercial value of the extended trading period, thus resulting in the business choosing not to use the hours it had obtained.

There were also concerns from residents’ representatives that the Review and appeals processes, lauded by government as major contributors to local democracy, still discouraged participation by members of the public due to lapses in the system of notification and the need to present oral evidence before a council chamber, or court of law, which many people found intimidating (Hadfield, 2006). Moreover, there were concerns that public sector professionals were often dismissive of residential complaints, especially if this threatened established understandings, or working relationships with the trade. Licensees seen to be cooperating with regulators through Pubwatch, for example, were suspected as having significant bargaining power over the treatment they received from licensing authorities, while there was no guarantee that residents could call upon police to support their representations, for example, against a Pubwatch member’s application to extend the hours of his or her licence. More broadly, our focus group with residents revealed deep-seated concerns over the extent to which their views on local licensing issues were taken into account by the authorities, particularly the police. Despite possessing formal statutory rights as ‘interested parties’, residents expressed frustration over the ways in which the informal instrumental relationships and bureaucratic imperatives which characterized local governance over the night tended to thwart opportunities for community mobilization.

### Strategic governance of the night-time city

Strategic area-based approaches are intended to ensure that while responses to alcohol-related crime and disorder may be strong in dealing with events, they do not remain weak in dealing with processes. Under the Guidance to the 2003 Act ‘cumulative impact’ refers to the effects of a significant number of licensed premises concentrated in one area where there is evidence that the environmental impacts of all these premises, taken together, is undermining the licensing objectives. In zones designated for Cumulative Impact Policies (CIPs), the burden of proof for new Premises Licence or licence variation applications is reversed and applicants must address the licensing authorities’ concerns in their proposed Operating Schedules. More specifically, the applicant must show that the proposed operation is in some way ‘exceptional’ and will not therefore add to the problems which necessitated the policy. Given that this tier of regulation served to establish a barrier to market entry for new businesses, existing licensees in the designated zones were, it was widely suggested, content for CIPs to remain unchallenged. Local authorities, however, were coming under political pressure from residents to create new CIPs, or to extend the spatial and temporal reach of existing ones. These demands were not typically viewed by CDRP practitioners as local democracy in action, but rather as unwelcome and unwarranted external pressure which threatened to disrupt carefully constructed strategies, alliances and ways of working. The following quotation is illustrative:

My battle at the moment is with people who want the CIPs extended … Strangely enough, I get no pressure from the trade to drop them and I think that’s because, although their lawyers want the business, the trade are quite happy with the hours they’ve got. I mean, it’s also interesting that it’s improved the value of their licences. (Council licensing officer)

Professional reticence concerning ‘untutored’ lay demands for CIPs emerged partly in response to the need for robust and legally defensive policies that, in accord, required painstaking development both of their wording and the evidential bases (containing quantitative and qualitative data) underpinning them. Moreover, there needed to be strong political will to maintain the legal momentum required to defend them:

when it goes to appeal, the magistrates find themselves saying ‘well the residents want 11 o’clock and the stress area policy says 11 o’clock, but the applicants are saying one o’clock, so we’ll give them midnight as a sort of compromise’. In one city, I’m talking with a council that doesn’t even act if a representation comes in support of its own stress area policy. So it’s [the CIP] hardly worth the paper it’s written on. (National civic amenity group activist)

Some CIPs were worded in such a way as to discourage applications only from certain ‘types’ of licensed premises that are commonly associated with greater risk of disorder, such as the ‘high volume vertical drinking establishment’. As understandings of what might constitute such premises remain ill-defined, the city’s CIP could easily be sidestepped:

If they come up with all the crime prevention measures I want, I don’t object to them. So you haven’t effectively stopped these areas from getting new premises … I think if it comes to it, they’ll remove the furniture and they’ll pack people in. Then, if they start to cause us problems we’ll take them to Review. But once they’ve got the Premises Licence, it’s too late effectively … the cumulative impact thing is by the by. (Police licensing officer)

The above reflections illustrate the challenge regulators face in attempting to address issues of ‘criminogenic process’ through legal mechanisms designed to preserve individual rights, such that each application must be judged on merit.

While CIPs were generally regarded as a useful tool for what one council officer described as ‘freezing the situation’; that is, preventing further environmental degradation, other mechanisms were seen as more suitable for reducing existing problems. ADZs provide a further strategic tool for governing nightlife settings, like CIPs, being based upon the view that it is not always possible to identify a clear relationship between crime and disorder occurring in the public realm and specific licensed premises, as offenders may have visited several premises in the course of an evening. ADZs aim to generate ‘improvements’ on an area-wide basis through the placing of a mandatory levy on licensed operators for the costs of CDRP-initiated crime control measures; levies are to be calculated individually for each premise within the designated zone in accordance with their rateable value and hours of operation.

Although operational in law for almost a year, no area has so far (as of June 2009) seen fit to impose an ADZ. This may be due, in part, to the accompanying guidance (Home Office, 2008) which frames the creation of an ADZ as an action of ‘last resort’ to be used only where other remedies have failed. It may also have to do with the instrumental fear of robust legal challenge from the national bar chains. Accordingly, in our research sites, ADZs were discussed with limited enthusiasm by interviewees from all sides of the alcohol policy debate. Much of this reluctance stemmed from the expressive qualities of the policy, specifically the assumed symbolic power of the label to define an area in terms of its high crime rate. Identifying a drinking circuit, or an entire town centre, as a hot-spot for disorder was thought to convey clear messages about the nature of risk and social relations within such places. This, it was feared, might deter ‘law-abiding’ people from visiting the area who may otherwise have contributed to enhanced levels of informal social control—an assumption supported by research concerning the socially mediated perceptual bases of ecological classifications and spatial difference (Sampson, 2009). While making the area less attractive to the legitimate visitor, it was thought likely to have the further effect of drawing in a disreputable contingent intent on adding to the area’s existing problems.

Before initiating an ADZ, Home Office guidance encourages CDRPs to work with licensees in drafting an area-wide ‘action plan’ which might involve asking for voluntary financial contributions towards initiatives such as taxi marshals and patrols by uniformed council ‘street ambassadors’. However, if the broader governmental objective is to lever resources from the private sector to fund additional services (policing or otherwise) within nightlife areas then their emergence seems likely to be usurped by the introduction of Business Improvement Districts (BIDs). The Local Government Act 2003 and the Business Improvement Districts (England) Regulations 2004 permit the development of private sector-led partnership initiatives in the form of BIDs, which are financed by a levy upon businesses and aspire to create a ‘safer trading environment’. BIDs differ from ADZs in that it is the fee payers, rather than the regulatory authorities, who establish and control the initiative. In November 2007, Nottingham city centre became the first area in the UK to create a BID scheme committed solely to the needs and priorities of the night-time economy.

## Discussion

It was clear from our interviews that premises-directed regulatory and enforcement activity was regarded as an interactive process in which the authorities entered into negotiation with the regulated and with interested parties. Powers—and especially, strategic area-directed powers—were used sparingly, their main utility being as levers or incentives for change, which encouraged compliance even if rarely directly applied with force (Braithwaite, 2002). Notions of the ‘attitude test’ in relation to the application of coercive powers were as pertinent here as in classic portrayals of the street policing encounter (Van Maanen, 1978). In dealing with licensed premises, especially the national chains, CDRPs thus applied a form of ‘smart power’, involving a judicious combination of hard (coercive) and soft (co-optive) elements (Nye, 2004).

Interviewees within CDRPs referred to the communicative properties of ‘tough laws’ and ‘clampdowns’ in demonstrating to the public that ‘something was being done’, yet were skeptical of centrally-driven enforcement campaigns due to the time-limited nature of the prioritization and additional resources. Several interviewees referred to parental ambivalence over alcohol, for instance, parents sending mixed-messages around their own drinking practices and the expectations they placed upon their children. Practitioners were conscious of their incapacity to deal with the issue of alcohol’s availability within the home and of the need to work with parents to discourage the purchase of drinks for their children. One informant referred to the development of local action to formalize such interventions through the use of Parenting Contracts, or Parenting Orders.

In relation to individual offenders—primarily, the disorderly ‘binge drinker’—the inability of punitive, generalized and spatially-oriented anti-social behaviour powers to nurture *pro-social* protective behaviours emerged as a major theme. The chronic necessity for police to focus on crowd control, public order maintenance and visible reassurance in nightlife zones at peak times resulted in limited capacities for arrest and routine proactive visits to licensed premises. Thus, notions of effectiveness were linked to the degree to which enforcement actions could be sustained and combined with other forms of targeted action in the long-term. Recent Home Office-instigated ‘crackdowns’ utilizing such powers were widely seen as ‘sticking plaster’ approaches (Crawford and Lister, 2007), most effective in tending the symptoms of a distempered social order, but ill-equipped to promote its systemic recovery. Our interviewees saw aspects of youth culture and wider societal attitudes to alcohol as providing positive endorsement for hedonistic, sometimes aggressive, drunken comportment; thus presenting stubborn root causes. Images of ‘urban grit’ (Talbot, 2007), the spectacle of crime (Tomsen, 1997), and tales of ‘daring do’ (Grazian, 2008) continued to stoke the fires of the NTE’s perennial appeal. These euphoric, almost celebratory elements of youthful nightlife culture are perfectly captured in the lyrical street poetry of Sheffield rock band, *The Arctic Monkeys*. Noting the desired and recreational elements of violence in which some of the night’s denizens ‘secretly … want it all to kick off, they want arms flying everywhere and bottles as well’ the band (in their late-teens at the time the song was written) highlight a crucial dichotomy in the framing of social interaction that is typical of everyday distinctions between night and day: ‘This town’s a different town today. This town’s a different town to what it was last night. You couldn’t have done that on a Sunday’ (*The Ritz to the Rubble*).

The ambiguous and hyper-vigilant (in some cases, arguably hypocritical) public and political sensibilities around alcohol being as they are, one problem faced by regulatory authorities was that of trying to reduce alcohol-related harms associated with the NTE without being drawn into the stigmatizing rhetoric within which much debate about young peoples’ drinking was framed. Tensions were heightened in local communities as a result of the normative aspects of the alcohol policy agenda and the clash of lifestyle, values and meaning attribution which constituted the contestation of urban space. The following views, expressed within our local residents’ focus group, are illustrative of such tensions:

They don’t think that people are sleeping, they don’t care that people are sleeping, and nobody is controlling that particular bit of garden. So they think, ‘hey, we’re free, we can do what we like’. And they do because they’re young people who are drunk.

Working with the licensed trade to limit ‘irresponsible’ supply was therefore regarded as necessary, but not sufficient. Some powers such as the ASBO were distinct in their emphasis on future conduct and an expansion in the use of contract and conditionality. Consumers could be taught ‘care of the self’ (Foucault, 1986) through health risk-focused and social norms education, thus stemming their demand for alcohol. Yet, summary disposals such as the PND provided little scope for engagement with the welfarist/disciplinary aspects of the criminal justice system, wherein ‘conditional cautioning’ and ‘alcohol arrest referral’ schemes acted as gateways into treatment services designed to help people ‘straighten out their lives’. Some respondents cautioned, however, that anti-social behaviour powers and premises-focused action must remain to the fore in providing rapid response to the distinct pressures that arose in locations where visitor numbers vastly outstripped those of the residential population toward which any longer term educative approaches might be directed.

Different social groups had varying capacities for choice and negotiation of the sometimes complex relations of inclusion and exclusion that operated in nightlife settings (Hadfield, 2008, 2009a; Marlière, 2007; Measham and Hadfield, in press; Moloney et al., 2009; Talbot, 2007). These processes had both formal and informal constituents, the former notably including the work of door staff and the expansion of surveillance technologies such as Identity Scanners at venue entrances, whilst the former related to the widely divergent identities, lifestyles and socio-economic status of co-present individuals and groups (Hadfield and Measham, 2009a). Thus, problems of order in night-time public spaces were generated, in part, by the fact that not all of those attracted by the ‘bright lights’ of the city found a welcome from licensed premises, not all of those welcomed by licensed premises found a welcome from the police or local residents, while relations between different groups of visitors could themselves be fraught or antagonistic. Place-based powers sought to respond to such governmental challenges as key components of a new ‘toolkit’ of urban control which favoured pre-emptive action, socio-spatial demarcation, and the embedding of proscribed consumption patterns and behavioural norms (Crawford, in press). This was not, however, simply the latest instalment of efforts to control the socially excluded ‘dangerous classes’. Temporality mattered, not only in relation to the unruly life of night but as a result of its political economy: drunken comportment was fuelled by the bonus and giro cheque alike, both providing the lifeblood of an important and established leisure economy.

In highlighting the attempt to remove disorder from key day-time citadels of consumption (notably, the shopping centre) liberal urbanist scholars have failed to account for the increasing centrality of such night-time consumer locales. Here one sees a gulf between the cultural norms of the night-time high street’s core constituency (and thus, the ‘included’, as well as the ‘the excluded’) and those of control agents. Any notions of the purification of urban public space and police potency in relation to such settings, should, we argue, be regarded as mythical. To assume that powers are applied and laws enforced, simply because they exist, is to grossly oversimplify the field of urban security governance. Empirical investigation reveals such governance to be characterized by subtle organizational and interpersonal power-plays which result directly, in the case of the NTE, from the contested nature of the field (Dennis and Martin, 2005). The state and its local ‘statutory authorities’ do not hold all the cards in the regulatory game, nor could they ever hope to do so given the social and economic complexities of the contemporary nightscape.

Notwithstanding the above, current levels of public, media, and political concern about alcohol continue to create a highly sensitized policy arena in which new powers are allocated to local statutory partnerships such that they may mould nightlife as never before. Controls are applied differentially, not only across areas, but also according to types of venue and social scene, as variously manifested in substance use preferences, consumer behaviours/expectations, and commercial business models (Graham and Homel, 2009; Hadfield, 2004, 2008; Hadfield and Measham, 2009). CDRPs are actively promoting and facilitating certain forms of leisure/cultural activity, while at the same time adjusting, restraining or banishing others. Examples of the latter include the City of Westminster’s ‘lighter touch’ approach to the licensing of restaurants, while the former is exemplified by Devon and Cornwall Constabulary’s use of s.160 Closure Orders to prevent the hosting of outdoor music festivals.

Developments on the horizon include the Policing and Crime Bill 2009 (currently in Lord’s Committee Stage) which proposes the introduction of a set of national ‘mandatory licensing conditions’ for licensed premises and ‘discretionary local licensing conditions’ that can be applied to groups of two or more premises in any given area (Home Office, 2009)**.** The Bill also seeks to amend police powers to deal with young people drinking alcohol in public. If passed, it will create a new offence of ‘persistently possessing alcohol in a public place’. Young people under 18 can be prosecuted for this offence if they are caught with alcohol in a public place three times within a 12 month period. The maximum punishment for this will be a level-two fine (currently £500). In addition, the Bill proposes that lap-dancing venues—rarely associated with disorder—are to be legally re-classified (subject to licensing authority designation), bringing them under the remit of the more stringent laws governing Sex Encounter Establishments (Hadfield and Measham, 2009; Hubbard, 2008).

Concerns arise in relation to ways in which the skewing of crime policy towards the most visible manifestations of Britain’s ‘alcohol problem’ may be permitting a more general expansion of control over the actions of those who occupy the public realm across a range of social settings and contexts. As in other areas of anti-social behaviour policy, the heaviest price in terms of criminalization is exacted upon those who have the least ability to resist the label: young people who procure public spaces for informal assembly, drinking and socializing and are ripe for repression in this respect, in Britain, as elsewhere (Basanta, 2009; Crawford, 2009; Selmini and Nobili, 2009). As Crawford (2007) notes, regulation is much more likely to prove ‘responsive’ to the needs and wishes of the regulated in the case of big business, much less so when dealing with individuals. Yet, outcomes vary. Despite central government attempts to steer and standardize their use, anti-social behaviour powers and their accompanying guidance are filtered and reinterpreted by local criminal justice practitioners and administrative bodies for whom there have been significant consequences in terms of resources, capacity building and professional skills development. Responses to crime and disorder are thus multi-faceted, diffuse, and differentiated, often involving significant degrees of locally-directed responsibilization and third-party enrolment (Hadfield, 2009b).

The joys and ills of contemporary nightlife raise key questions about the conditions necessary to achieve civil and diverse urban spaces; put differently, where are we to find the ‘social’ in the debate on anti-social behaviour in our towns and cities after dark when it proves so difficult to even achieve the co-presence of different generations and communities? Have the optimistic visions of a more convivial NTE so characteristic of public debate a decade hence now evaporated into grim cynicism concerning a nation’s problematic relationship with alcohol? The post-2003 Licensing Act context is a complex one in which the art of urban security governance involves attempts to balance the seductions of the market, consumer freedoms and civil liberties, with surveillance, securitization and repression. Whilst the relative power of the various stakeholders may ebb and flow, it seems likely to remain ever thus.
